# Smart Grid Stability Prediction Model Using Neural Networks to Handle Missing Inputs

**DOI:** 10.3390/s22124342

**Published:** 2022-06-08

**Authors:** Madiah Binti Omar, Rosdiazli Ibrahim, Rhea Mantri, Jhanavi Chaudhary, Kaushik Ram Selvaraj, Kishore Bingi

**Affiliations:** 1 Department of Chemical Engineering, Universiti Teknologi PETRONAS, Seri Iskandar 32610, Malaysia; madiah.omar@utp.edu.my; 2 Department of Electrical and Electronics Engineering, Universiti Teknologi PETRONAS, Seri Iskandar 32610, Malaysia; rosdiazli@utp.edu.my; 3 School of Electrical Engineering, Vellore Institute of Technology, Vellore 632014, India; rhea.mantri@gmail.com (R.M.); jhanavi0513@gmail.com (J.C.); kaushikram0100@gmail.com (K.R.S.)

**Keywords:** four-node star network, feedforward neural network, forecast, prediction, smart grid, stability

## Abstract

A smart grid is a modern electricity system enabling a bidirectional flow of communication that works on the notion of demand response. The stability prediction of the smart grid becomes necessary to make it more reliable and improve the efficiency and consistency of the electrical supply. Due to sensor or system failures, missing input data can often occur. It is worth noting that there has been no work conducted to predict the missing input variables in the past. Thus, this paper aims to develop an enhanced forecasting model to predict smart grid stability using neural networks to handle the missing data. Four case studies with missing input data are conducted. The missing data is predicted for each case, and then a model is prepared to predict the stability. The Levenberg–Marquardt algorithm is used to train all the models and the transfer functions used are *tansig* and *purelin* in the hidden and output layers, respectively. The model’s performance is evaluated on a four-node star network and is measured in terms of the MSE and R${}^{2}$ values. The four stability prediction models demonstrate good performances and depict the best training and prediction ability.

## 1. Introduction

The conventional power grid contains standard power generation units grounded on fossil fuels. With the soaring energy prices, the need for renewable energy sources and climate change, the old power grid is becoming outdated and facing various limitations, such as cybersecurity, privacy and power losses due to one-way communication [1]. This pushes for deploying renewable energy sources to improve sustainability and reliability. A smart grid is a solution to this. The smart grid system is a digital future electricity system that enables a two-way flow of communication, i.e., between the center and the device to the center [2].

This bidirectional communication utilizes advanced computing infrastructure, digital sensing and software capabilities to optimize all the grid components and improve reliability and sustainability. There is a unidirectional flow of energy from the energy provider to the consumer in a traditional grid, and consumers are charged based on their consumption. However, in a smart grid system, the users in the grid can consume, produce, store and trade energy with other users [3]. The smart grid introduces demand response, and the price information is determined as the demand is evaluated with supply and conveyed to the customer.

This paper used the DSGC model to define and relate the price to the grid frequency [2,4]. The mathematical model based on DSGC differential equations seeks to find grid instability for a four-node star architecture [5]. The four-node star architecture consists of a central generation node, the power source and three consumer nodes. The response time of the smart grid users is considered to adjust the consumption/production concerning the price changes.

The model involves real-time pricing, and thus the grid stability has to be maintained with fluctuations in reaction times and electricity price of all users. It is critical to evaluate grid stability as the process is time-critical dynamically. This is because smart grid stability prediction helps increase efficiency through grid optimization, improves electrical supply reliability and consistency and analyses disturbances and fluctuations in energy consumption or production.

Before the utilization of modern techniques to predict smart grid stability, traditional approaches consisted of simulations combining fixed values for one subset and fixed distribution of values for the remaining subset variables [6,7]. The generation of electricity by photovoltaic power is related to the global horizontal irradiance. For the unknown cloud statistics, the irradiance is uncertain for predicting the stability in power generation, causing optical instability in the solar irradiance [4]. Measurement-based methods are another complex and challenging traditional method used to predict power grid stability [8].

Various statistical approaches have been investigated, including autoregressive moving average, Kalman filter and Markov chain model [9], which contribute to insufficient reliability of the grid [10]. Other types of early statistical methods [11] for load forecasting in smart grids have various drawbacks and affect the accuracy of the prediction model. These are built by ineffective, simple regression functions, and thereby do not yield good performance in vast uncertainties [12]. Further, traditional approaches, such as time series analysis, ARMA, ARIMA and Markov models for stability forecasting, exist only in specific operating ranges [13,14].

Additionally, some research involved using conventional parametric methods that include linear regression, auto-regressive moving average and the general exponential methods. Although such models return satisfactory prediction accuracy, they persist with major disadvantages, such as improper response and complex computational problems to meteorological variables and nonlinear electrical load [15]. A probabilistic model was introduced in [16] for power stability.

However, some uncertainties have been observed between regular grid operation and cascading failure operation in the simulation result. Adding on, techniques used for stability assessment require extensive computation time and massive data analysis volume, which makes it tough to obtain a reliable prediction and makes it difficult to take decisions for an operating power system [17].

A few hybrid systems used for dynamic stability prediction have been based on unreliable self-organized maps and responded slowly [6,18]. Another method introduced a situational awareness for stability prediction, a perception of elements for a given time and space in the environment [5]. It was proven that optimized deep-learning models are one of the excellent prediction tools for smart grid stability. Using neural networks for stability prediction has various advantages.

They have multiple training algorithms, do not require significant dataset pre-processing and can produce high accuracy values during training and testing [4]. Further, they can recognize different sets within a whole dataset and give adequate results even when the dataset is incomplete or inaccurate [19]. Finally, the ability to implicitly detect complex nonlinear relationships between independent and dependent variables makes it viable for stability prediction [20].

Comprehensive review work in [21] concluded that most of the works on prediction models using machine learning reported little or no information on the presence and handling of missing data. The missing data is omitted in most models, which is ineffective, affecting their performance. The missing data for the analysis results from many things, such as sensor failure, equipment malfunctions, lost files, etc. This challenges the increasing cost and prediction ability of the proposed models. Thus, there is a need for significant research in handling missing data. On the other hand, predicting the data with neural networks or machine-learning models is more efficient than simply omitting the data or resorting to mean values.

Being motivated by the literature, this paper proposes a novel method to predict the smart grid stability of a four-node star network using a neural network with complete and missing input data, consisting of missing input variables. Thus, the significant contributions of this paper are highlighted as follows:

- The classic FFNN is designed to predict the stability of the smart grid system of a four-node star network with complete input data.
- The sub-neural networks are proposed to predict the missing input variables, which are caused due to a sensor, network connection or other system failures. Then, the system’s stability is forecast using these predicted missing input data.
- The performance of the proposed approach is evaluated in four different case studies in which at least one input variable is missing.

The subsequent sections of the paper are organized as follows: Section 2 presents the comprehensive literature review on smart grid stability prediction using neural networks. Section 3 describes the mathematical modeling and data description of the four-node star network used for the smart grid stability prediction. Section 4 shows the development and performance of the FFNN with complete input data, and Section 5 describes the development and performance evaluation of the FFNN to handle the missing inputs. Finally, Section 6 highlights the conclusions of the proposed work.

## 2. Literature Review

In this section, an extensive literature survey is conducted on smart grid stability using neural networks. This literature review shows that various neural-network-based techniques have been used for analysis, and the data analyzed in the works are with complete input data. The developed approaches are robust and accurate due to their complex structure that helps classify problems and recognize correlations in raw data and hidden patterns.

**Figure 1:**
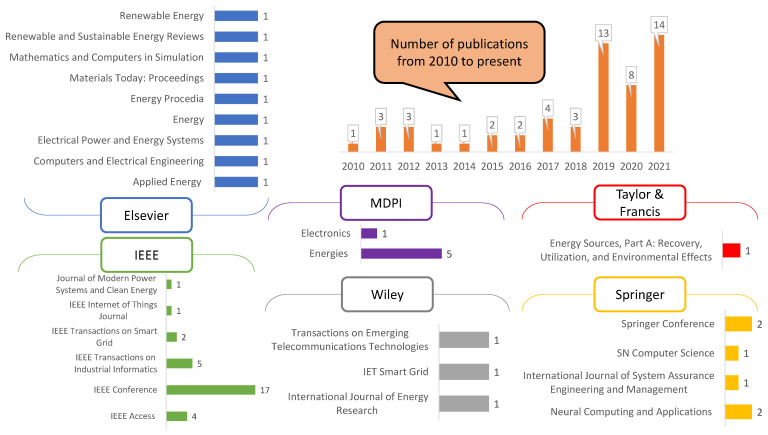
Year-wise and the publisher-wise contributions to smart grid’s stability forecasting during the last decade.

**Figure 2:**
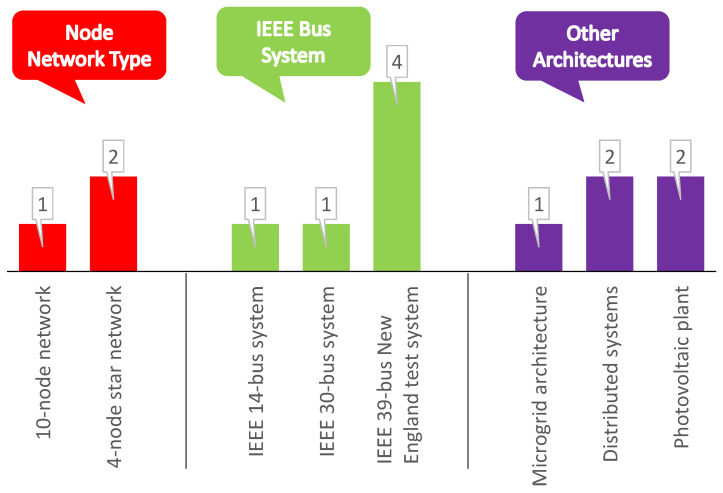
Summary of the smart grid architectures identified in the conducted literature survey.

A summary of works focused on smart grid stability prediction using various neural networks is highlighted in Table 1. The table contains 55 papers published in the last decade, categorized into publication year, smart grid architecture, neural network type, neural network architecture, activation functions, training algorithms, performance measures and comparison techniques considered for each study. From the research works in Table 1, the year-wise and the publisher-wise contributions to the smart grid’s stability prediction during the last decade are shown in Figure 1. Figure 2 depicts the smart grid architectures identified in the literature survey conducted.

The most popular architectures are IEEE bus systems [6,16,18,22,23] and node network types [4,8,24]. Therefore, in this paper four-node star network was selected to perform the proposed research.

**Figure 3:**
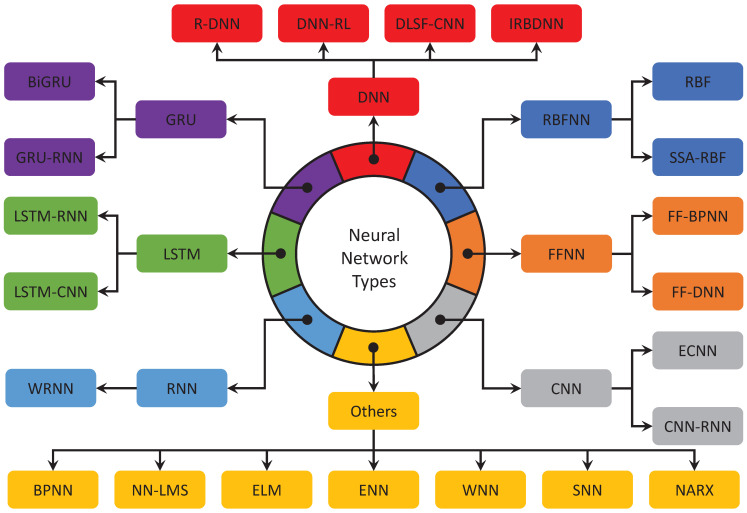
Classification of multiple neural-network-based models developed for smart grid stability prediction.

In the analysis, several types of neural networks and hybrid networks were identified, as depicted in Figure 3. Among the most popular neural networks identified are FFNN, which includes the hybridized versions, such as FF-BPNN [25] and FF-DNN [26]. In addition, CNN is another most widely used, with its enhanced and hybrid versions, namely ECNN [27] and CNN-RNN [28]. The hybrid versions of LSTM, including LSTM-RNN [29,30] and LSTM-CNN [31], can also be seen in this literature.

In addition, the performance of DNN for stability prediction was improved by hybridizing with RNN, RL [32], CNN and IRBDNN [33]. On the other hand, optimization algorithms, such as SSA, have also been used with RBFNN to obtain the network’s optimal weights [7]. The hybrid versions of GRU models, such as BiGRU [8] and GRU-RNN [9], have also been used for node networks’ stability prediction. The sub-classification of all these neural-network-based models is also illustrated in Figure 3.

**Figure 4:**
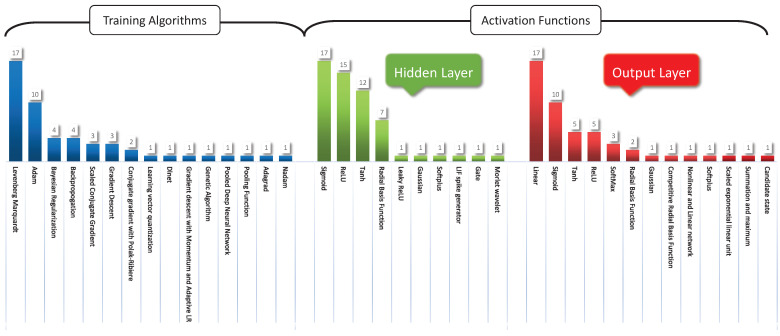
Summary of the various training algorithms and activation functions used in neural-network-based models for smart grid stability prediction.

Figure 4 summarizes the various training algorithms and activation functions used in the research work reported in Table 1. The figure concludes that the LM algorithm is the most commonly used for training algorithms, followed by Adam’s optimization algorithm [29,32]. Furthermore, it further depicts that sigmoid, ReLU, *tansig* and *tanh* are the most frequently used hidden layer activation functions [30,34]. In contrast, the *purelin* activation function followed by Sigmoid is most commonly used in the output layer of the neural network [34,35,36].

The significant findings from the literature review on smart grid stability prediction using neural networks are highlighted as follows:

- No work was conducted to predict stability when there is a missing parameter. Most studies showed that missing data had been either omitted, unreported or replaced with mean/median values.
- The most popular architectures used for the case studies are IEEE bus systems and node network types (see Figure 2).
- Among the several types of conventional and hybrid neural networks proposed in the literature, the FFNN and its hybrid versions, such as FF-BPNN and FF-DNN, are widely presented (see Figure 3 and Table 1).
- The Levenberg–Marquardt algorithm is the most frequently used training algorithm for various networks to predict smart grid stability (see Figure 4).
- The *tansig* and *purelin* activation functions have frequently been used in various networks’ hidden and output layers to predict smart grid stability (see Figure 4).

From the above research gaps, this paper made an effort to develop a forecasting model that handles the missing input data. For the proposed neural-network-based forecasting model, the LM training algorithm was selected as it is one of the fastest backpropagation algorithms and is widely recommended in the literature. The literature proves that effective training necessitates a nonlinear and linear combination of activation functions. Thus, the *tansig* and *purelin* activation functions are utilized in the hidden and output layers, respectively.

Further, in our previous work reported in [37], an effort was made to compare the performance of FFNN, cascade and recurrent neural-network-based models for smart grid stability prediction. The work concludes that, for the considered application, the FFNN demonstrated superior performance in terms of the MSE and R${}^{2}$ values compared to cascade and recurrent neural networks. On the other hand, over the years, researchers have proposed different methodologies and theories for selecting the number of hidden layers and the number of hidden neurons in each hidden layer. As reported in [38], it was concluded that a network with only one hidden layer but sufficient neurons can achieve better performance.

Moreover, this performance can be further improved by adding additional hidden layers. However, the variation in this performance with a multilayer network is minimal. The work reported in [39] concluded the same, stating that a multilayer network has achieved better performance but increased the complexity of the network. Therefore, for the considered application, the FFNN with a single hidden layer was used for all the cases, which improved the performance in predicting smart grid stability.

**Table 1:** Summary of works focused on forecasting smart grid stability using neural networks.

Ref.	Year	Smart Grid Architecture	Neural Network Type	Neural Network Architecture	Activation Functions		Training Algorithm	Performance Measures	Comparison Techniques
					Hidden Layer	Output Layer			
[34]	2021	–	FFNN	2:10:1	Tanh, Sigmoid	Linear	LM, BR, SCG	MSE, R	RTP, SMP, RTP-SMP, GA, ANN, STW
[7]	2021	Smart grid with photovoltaic and wind turbine	SSA-RBFNN	–	–	–	–	RMSE	SSA-RBFNN with and without RES
[40]	2021	–	FFNN	3:20:1	Sigmoid	Linear	LM	MSE, RMSE	PV with ANN, Wind with ANN, Hybrid model with ANN
[32]	2021	–	DNN-RL	–	Leaky ReLU	Leaky ReLU	Adam	MSE	–
[29]	2021	–	LSTM-RNN	1:50:50:50:1	Tanh	Tanh	Adam	MAE, RMSE, MAPE	GBR, SVM
[4]	2021	four-node star	FFNN	24:24:12:1	ReLU	Sigmoid	Adam, GDM, Nadam	Accuracy, Precision, Sensitivity, F-score	CNN, FNN
[9]	2021	–	GRU-RNN	3:15:10:1	Gate	Candidate	AdaGrad	RMSE, MAE	LSSVR, WNN, ELM, SAE, DBN
[41]	2021	–	SNN	784:400:400:11	LIF spike generator	Summation and maximum	–	Precision, Recall, F-score, Accuracy	CNN
[8]	2021	four-node star	LSTM, BiGRU, ELM	12:256:128:1, 12:512:256:1, 12:96:30:1	Sigmoid Softplus	Sigmoid Softplus	Adam	RMSE, MAE, R${}^{2}$, PICP, PINC, ACE	BiGRU, LSTM, XGB, LGBM, ANN
[42]	2021	Distributed systems	DNN-RL	–	ReLU	ReLU	Adam	Peak, Mean, Var, PAR, Cost, Computation time	C-DDPG, DPCS, SWAA
[11]	2021	–	LSTM, BPNN	6:96:48:1, 6:48:24:1, 6:10:1	RBF	Sigmoid	Adam	MAPE, RMSE	LSTM, BPNN, MLSTM, ELM, MLR, SVR
[43]	2021	–	BPNN	3:2:3	Sigmoid	Linear	BP	RMSE	–
[14]	2021	–	FF-DNN	–	ReLU	SELU	PDNN, Pooling function	FA, MAE, RMSE, SoC, HR	SVM, NN-ARIMA, DBN
[44]	2021	–	FFNN	–	ReLU	Alpha	BP	Accuracy, Precision, Recall, F-score	PSO-KNN, PSO-NN, PSO-DT, PSO-RF
[10]	2020	–	CNN-LSTM	–	ReLU	Linear	Adam	RMSE, MAE, NRMSE, F-score	ARIMA, BPNN, SVM, LSTM, CEEMDAN-ARIMA, CEEMDAN-BPNN, CEEMDAN-SVM
[45]	2020	–	RNN, CNN	–	Sigmoid	Tanh	Adam	Area under the curve, F-score, Precision, Recall, Accuracy	Logistic regression, SVM, LSTM
[46]	2020	–	NN-LMS	24:24:24, 24:96:96:4	ReLU	ReLU	–	–	–
[47]	2020	–	NARX-RNN	2:5:1	Sigmoid	Linear	Conjugate gradient with Polak-Ribiere	NRMSE, RMSE, MAPE	ARMAX
[48]	2020	–	FFNN	20:38:1	Tanh	Linear	Conjugate gradient with Polak-Ribiere	MSE	RTEP, LBPP, IBR without ESS
[33]	2020	–	IRBDNN	–	–	–	–	RMSE, MAE, MAPE	DNN, ARMA, ELM
[30]	2020	–	LSTM-RNN	–	Sigmoid, Tanh, ReLU	–	–	Accuracy, Precision, Recall, F-score	GRU, RNN, LSTM
[22]	2020	IEEE 14-bus system	CNN	–	ReLU	Sigmoid	Adam	Precision, Recall, F-score, Row accuracy	SVM, LGBM, MLP
[49]	2019	–	FF-BPNN	–	–	–	GA	MSE, Fitness, Accuracy	–
[50]	2019	–	RNN		Tanh	Sigmoid	BP	MAE, RMSE, MAPE, Pmean	BPNN, SVM, LSTM, RBF
[28]	2019	–	CNN-RNN	100:98:49:1	ReLU	Softmax		MSE, Recall, PTECC	CNN, CNN-RNN, LSTM
[51]	2019	–	ENN	10:1:1	–	–	GDM and Adaptive LR, LM	RMSE, NRMSE, MBE, MAE, R, Forecast skill	Similarity search algorithm, ANN, MLP and ARMA, LSTM
[12]	2019	–	FF-DNN, R-DNN	2:5:2	Sigmoid, Tanh, ReLU	Sigmoid, Tanh, ReLU	LM	MAPE	Ensemble Tree Bagger, Generalized linear regression, Shallow neural networks
[31]	2019	–	CNN, LSTM	05:10:100	ReLU	Softmax	–	MCC, F-score, Precision, Recall, Accuracy	Logistic regression, SVM
[27]	2019	–	ECNN	32:32:1	ReLU	Sigmoid, Softmax	Adam	MAE, MAPE, MSE, RMSE	AdaBoost, MLP, RF
[23]	2019	IEEE 39-bus New England test system	CNN, LSTM	–	Sigmoid	Tanh	GDM	Accuracy	–
[52]	2019	–	FFNN	76:20:1, 92:20:1, 92:20:1	ReLU	Sigmoid	LM	MSE, Accuracy, Precision, Recall, F-score	RF, OneR, JRip, AdaBoost-JRip, SVM and NN (without WOA)
[53]	2019	–	ECNN	–	–	–	–	MSE, RMSE, MAE, MAPE	
[54]	2019	–	FF-DNN, R-DNN	–	Sigmoid, Tanh, ReLU	Linear	LM	MAPE, Correlation coefficient, NRMSE	ANN, CNN, CRBM, FF-DNN
[25]	2019	–	FF-BPNN	–	–	ReLU	GDM	Mean error, MAD, Percent error, MPE, MAPE	Classical forecasting methods
[26]	2019	–	FF-DNN	1:5:1, 6:5:1	Sigmoid	Linear	–	MAPE	DNN-ELM
[55]	2018	–	FFNN	–	Sigmoid	Nonlinear and linear network	LM	MSE, R	Multilayer ANN Models
[56]	2018	–	RBF, WRNN	7:4:3	RBF	Competitive	LM	Classification accuracy	Pooling Neural Network, LM
[13]	2018	–	WRNN	2:16:16:4	RBF	RBF	–	RMSE	–
[57]	2017	–	FFNN	7:96:48:24:1	Tanh	Gaussian	Dlnet, BP	MAPE	Ten state-of-the-art forecasting methods
[58]	2017	–	FFNN	24:5:1	Sigmoid	Sigmoid	LM	MAPE	AFC-STLF, Bi-level, MI-ANN forecast
[59]	2017	–	Deep learning based short-term forecasting	20:30:25:1	ReLU	ReLU	–	RE	SVM
[24]	2017	10-node network	FFNN, WNN-LQE	8:10:1	Morlet wavelet	Sigmoid	–	SNR	LQE-based WNN, BPNN, ARIMA, Kalman, XCoPred algorithms
[60]	2016	–	FFNN	3:20:10:3	Sigmoid	Linear	LM, BR	MSE, R	LM, BR
[15]	2016	–	FFNN	8:10:1	Sigmoid	Linear	–	MAE, MAPE, RMSE, R${}^{2}$, MSE	GA-MdBP, CGA-MdBP, CGASA-MdBP
[16]	2015	IEEE 30-bus system	FFNN	4:10:1	RBF	–	SCG supervised learning	MSE, PDF, CDF	–
[61]	2015	–	FFNN	10:1:20	Tanh	Tanh	LVQ	Mean Error, Maximum Error, Success %	–
[62]	2014	–	FFNN	7:(10-15):1	Sigmoid	Linear	LM	R, MAPE	–
[17]	2013	–	FFNN	–	–	–	LM	MER, MAE, MAPE	–
[63]	2012	Microgrid architecture: residential smart house aggregator	BPNN	10:1:1	Tanh	Linear	LM, SCG	Solar insulation and air temperature	–
[64]	2012	IEEE 39-bus New England test system	FF-BPNN	20:10:5:1	Tanh	Sigmoid	LM, BR	Stability	–
[6]	2012	IEEE 39-bus New England test system	RBF	30:30:9, 30:30:10	RBF	Linear	LM	Training Time, Testing Time, Number of misses, MSE, Classification accuracy %	
[18]	2011	IEEE 39-bus New England test system	RBF	36:36:1	Gaussian	Linear		Training time, Testing time, Number of misses, MSE, False alarms %, Misses %, Classification accuracy %	Traditional NR method
[65]	2011	Grid-connected PV plant	BPNN	16:15:7:1	Sigmoid	Linear	LM	MABE, RMSE, R	–
[66]	2011	Medium tension distribution system	RBF	33:119:33, 33:129:33	RBF	Linear	–	MSE, SPREAD	–
[67]	2010	–	BPNN, FFNN	8:8:30:1	Tanh	Linear	LM, BR	MSE	LM, BR, OSS

## 3. Mathematical Modeling and Data Description of Four-Node Star Network

In Section 3.1, the mathematical modeling of the four-node star architecture network is developed based on the equations of motion and binding the electricity price to the grid frequency. Then, the description of the generated dataset from the final dynamic equation of DSGC and the correlation analysis between the network parameters are provided.

### 3.1. Mathematical Modeling and Stability Analysis of Four-Node Star Network

In this section, the mathematical modeling of the four-node star network and the stability analysis are conducted. The central node (center of the “star”) communicates directly with the consumer nodes in a star network topology. The consumer nodes are connected to the central (generation node), enabling bidirectional communication between each node, which helps them to operate at lower power levels. One of the main advantages of star topology is that the networks are independent. In case of failure or errors in one of the consumer nodes, the other consumer nodes are not affected, and the network operates typically.

**Figure 5:**
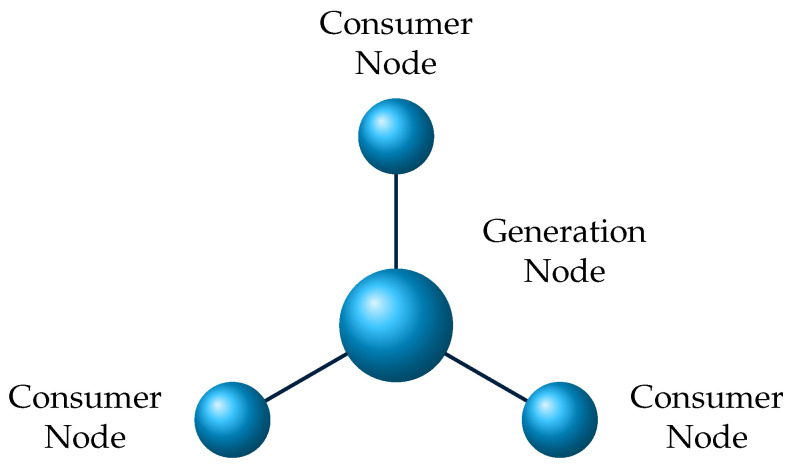
The architecture of the four-node star network.

The network is formed with one power producer in the center (i.e., generation node) and three consumers (i.e., consumer node). Star topologies depend heavily on delay and averaging time. Intermediate delays in a four-node star topology benefit stability, making it a simple, effective and efficient system [4]. From the literature survey conducted, we observed that the star and bus topologies were popular. A conclusion was drawn that star networks used in previous works having similar objectives showed good performance and can achieve the mathematical modeling for the DSGC system. Thus, the four-node star topology was chosen for this work, and the mathematical model of the DSGC system was obtained for the four-node star architecture network given in Figure 5.

#### 3.1.1. Mathematical Modeling

The mathematical model of the DSGC system is obtained for the four-node star architecture network given in Figure 5. The figure shows that the network is formed with one power producer in the center (i.e., Generation Node) and three consumers (i.e., Consumer Node). The mathematical modeling developed with assumptions, such as no uncertainties and external disturbances comprises two parts. The first describes the generator and load dynamics based on equations of motion. The second part is based on binding the electricity price to the grid frequency [4,5,37,68].

The first step in the modeling is applying the energy conservation law. As per the energy conservation law, the power balance equation is given as follows:

(1) $$P^{s}=P^{a}+P^{d}+P^{t},$$

where $P^{s}$ is the power generated from source.

In (1), $P^{d}$ is the dissipated energy from the turbine, which is proportional to the angular velocity square given as,

(2) $$P^{d}=K_{j}{\left({\dot{\delta }}_{j}\left(t\right)\right)}^{2},$$

where *j* is the node index (either generator or load), $K_{j}$ is the friction coefficient of jth node and $\delta_{j}\left(t\right)$ is the rotor angle of jth node defined as,

(3) $$\delta_{j}\left(t\right)=\omega t+\theta_{j}\left(t\right),$$

where ω is the grid frequency and $\theta_{j}$ is the relative rotor angle.

Similarly, in (1), $P^{a}$ is the accumulated kinetic energy, and $P^{t}$ is the transmitted power given as,

(4) $$P^{a}=\frac{1}{2}M_{j}\frac{d}{\mathrm{dt}}{\left({\dot{\delta }}_{j}\left(t\right)\right)}^{2},$$

(5) $$P^{t}=-\sum_{m=1}^{4}P_{jm}^{\max}\sin\left(\delta_{m}-\delta_{j}\right),$$

where $M_{j}$ is the moment of inertia of jth node and $P_{jm}^{\max}$ is the maximum capacity of line between jth and mth node.

By substituting (2), (4) and (5) in (1), $P_{j}^{s}$ is obtained as follows:

(6) $$P_{j}^{s}=\frac{1}{2}M_{j}\frac{d}{\mathrm{dt}}{\left({\dot{\delta }}_{j}\left(t\right)\right)}^{2}+K_{j}{\left({\dot{\delta }}_{j}\left(t\right)\right)}^{2}-\sum_{m=1}^{4}P_{jm}^{\max}\sin\left(\delta_{m}-\delta_{j}\right)$$

Now, substituting $\delta_{j}\left(t\right)$ from (3) in (6), $\frac{d^{2}}{{\mathrm{dt}}^{2}}\theta_{j}\left(t\right)$ is obtained as follows:

(7) $$\frac{d^{2}}{{\mathrm{dt}}^{2}}\theta_{j}\left(t\right)=P_{j}-\alpha_{j}\frac{d}{\mathrm{dt}}\theta_{j}\left(t\right)+\sum_{m=1}^{4}K_{jm}\sin\left(\theta_{m}-\theta_{j}\right),$$

where $P_{j}$ is the generated or consumed power, $\alpha_{j}$ is the damping constant and $K_{jm}$ is the coupling strength between jth and mth nodes. These coefficients are computed as follows:

(8) $$P_{j}=\frac{P_{j}^{s}-K_{j}\omega^{2}}{M_{j}},\;\alpha_{j}=\frac{2K_{j}}{M_{j}},\;K_{jm}=\frac{P_{jm}^{\max}}{M_{j}\omega }.$$

The final step in the modeling is binding the electricity price to the grid frequency ω, allowing consumers to adjust their consumption or production. Thus, the electricity price $p_{j}$ for the jth node is computed as,

(9) $$p_{j}=p_{\omega }-c_{1}\int_{t-T_{j}}^{t}\frac{d}{\mathrm{dt}}\theta_{j}\left(t-\tau_{j}\right)dt,$$

where $p_{\omega }$ is the electricity price when $d\theta_{j}/\mathrm{dt}=0$, $c_{1}$ is the proportionality coefficient, $T_{j}$ and $\tau_{j}$ are the average and reaction times, respectively.

The power consumed or produced ${\hat{P}}_{j}\left(p_{j}\right)$ at price $p_{j}$ is defined as,

(10) $${\hat{P}}_{j}\left(p_{j}\right)\approx P_{j}+c_{j}\left(p_{j}-p_{\omega }\right),$$

where $c_{j}$ is the coefficient proportional to the elasticity price.

For the four-node star network shown in Figure 5, it is assumed that the algebraic sum of power consumed or generated is equal to zero. Thus, the assumption is given as,

(11) $$\sum_{j=1}^{4}P_{j}=0.$$

Therefore, the final dynamic equation of DSGC system for the four-node star architecture network is obtained by substituting (7), (9) and (10) in (11) as follows:

(12) $$\frac{d^{2}}{{\mathrm{dt}}^{2}}\theta_{j}\left(t\right)=P_{j}-\alpha_{j}\frac{d}{\mathrm{dt}}\theta_{j}\left(t\right)+\sum_{m=1}^{4}K_{jm}\sin\left(\theta_{m}-\theta_{j}\right)-\frac{\gamma_{j}}{T_{j}}\left(\theta_{j}\left(t-\tau_{j}\right)-\theta_{j}\left(t-\tau_{j}-T_{j}\right)\right),$$

where $\gamma_{j}=c_{1}\times c_{j}$.

#### 3.1.2. Stability Analysis

In the first stage of analyzing the network’s dynamical stability around the grid’s steady-state operation, the fixed points of the network are computed by solving $\frac{d^{2}}{{\mathrm{dt}}^{2}}\theta_{j}=0$ and $\frac{d}{\mathrm{dt}}\theta_{j}=0$, which are obtained as,

(13) $$\left(\theta_{j}\left(t\right),\frac{d}{\mathrm{dt}}\theta_{j}\left(t\right)\right)=\left(\theta_{j}^{*},\omega_{j}^{*}\right).$$

The above equation shows that the fixed point exists only if the grid has an adequate coupling strength coefficient $K_{jm}$ to transmit the power from the generation nodes to the consumer nodes. Furthermore, in the obtained fixed point, the value of $\omega_{j}$ is $\frac{d}{\mathrm{dt}}\theta_{j}$, which is equal to zero. Thus, the value of $\omega_{j}^{*}=0$. The fixed points highlight that it only depends on the value of $\theta_{j}$, which must be analyzed to determine the stability.

Next, the Jacobian matrix of the system is obtained to compute the eigenvalues that determine the network’s stability. Thus, the Jacobian matrix *J* is calculated as,

(14) $$J=\left(\begin{array}{cc} \frac{\partial }{\partial \theta_{j}}\left(\frac{d}{\mathrm{dt}}\theta_{m}\right) & \frac{\partial }{\partial \omega_{j}}\left(\frac{d}{\mathrm{dt}}\theta_{m}\right)\\ \frac{\partial }{\partial \theta_{j}}\left(\frac{d}{\mathrm{dt}}\omega_{m}\right) & \frac{\partial }{\partial \omega_{j}}\left(\frac{d}{\mathrm{dt}}\omega_{m}\right) \end{array}\right).$$

The eigenvalues λ of the above Jacobian matrix determine the network’s stability. The matrix has infinitely many solutions. However, only a finite number of solutions can have a real positive component (Re(λ)≥0), determining the network’s instability. In addition, the negative real part (Re(λ)<0) indicates stability. Therefore, the network’s stability condition is summarized as follows:

(15) Stability=Stable,ifRe(λ)<0,Unstable,ifRe(λ)≥0.

### 3.2. Data Description of Four-Node Star Network

From the differential Equation (12), it is to be noted that the parameters $\tau_{j}$, $P_{j}$ and $\gamma_{j}$ are the predictive features of the network. The values of these parameters used for the simulation are shown in Table 2 [68]. The value of *j* ranges from 1 to 4, in which index 1 is the generator node, and the remaining indices (2, 3 and 4) are consumer nodes. Further, the values of simulation constants $\alpha_{j}$, $T_{j}$ and $K_{jm}$ used in the simulation are given in Table 2. The range of $P_{j}$ (j∈(2,3,4)) at the consumer nodes are also shown in Table 2. The value of $P_{1}$ at the generating node in the model is computed as,

(16) $$P_{1}=\sum_{j=2}^{4}P_{j}.$$

**Figure 6:**
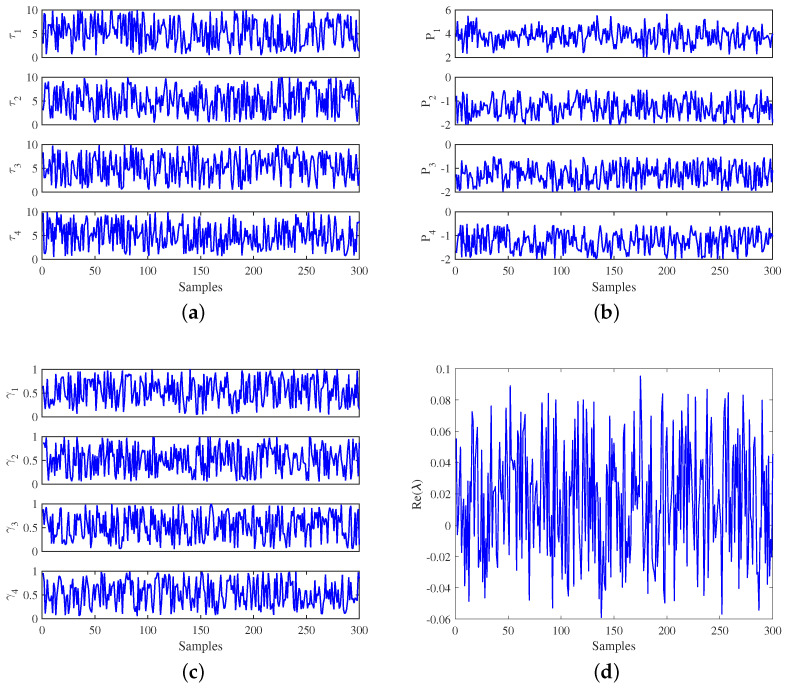
Dataset of predictive and dependent features of four-node star network. (**a**) Reaction time $\tau_{j}$; (**b**) Produced/consumed power $P_{j}$; (**c**) Elasticity coefficient $\gamma_{j}$; (**d**) Re(λ).

The generated dataset contains 60,000 samples for all the 12 predictive variables and one dependent variable, Re(λ). The predictive features are shown in Figure 6a–c, and the dependent variable Re(λ), whose values are the real part of the roots from the dynamic equation of DSGC system in (12), is shown in Figure 6d. The dataset is zoomed in, and the region of the first 300 samples is shown in the figures.

### 3.3. Correlation Analysis

**Figure 7:**
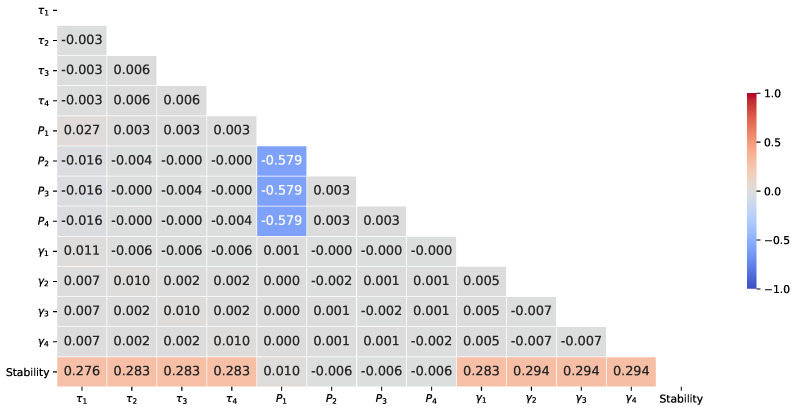
Pearson’s correlation matrix of the study variables.

The Pearson’s correlation matrix between the predictive features of the network ($\tau_{j}$, $P_{j}$, $\gamma_{j}$) and dependent variable Re(λ) is shown in Figure 7. As reported in [69], the interpretation from the Pearson’s correlation coefficients is given in Table 3. Therefore, from Figure 7 and Table 3, it can be observed that there is a moderate negative correlation coefficient of −0.579 between $P_{1}$ and its sum components ($P_{2}$, $P_{3}$ and $P_{4}$). In addition, there is a weak positive correlation between dependent variable Re(λ), $\tau_{j}$ and $\gamma_{j}$ of around 0.28 and 0.29, respectively. In contrast, there is negligible correlation between dependent variable Re(λ) and $P_{j}$. Furthermore, it is worth highlighting that there is a negligible correlation between the predictive features ($\tau_{j}$, $P_{j}$, $\gamma_{j}$) of the network.

As Pearson’s correlation matrix describes the strength and associated direction between the variables, it can be concluded that the relationship between any two predictive features or between predictive features and the dependent variable is not very strong. Therefore, in this analysis, all the parameters are considered for developing and evaluating the performance of the proposed model (refer to Section 4). In addition, only the power parameters have been considered for developing and assessing the performance of the proposed model that handles the missing inputs since there is a moderate correlation between $P_{1}$ and its sum components compared to other parameters (refer to Section 5).

**Figure 8:**
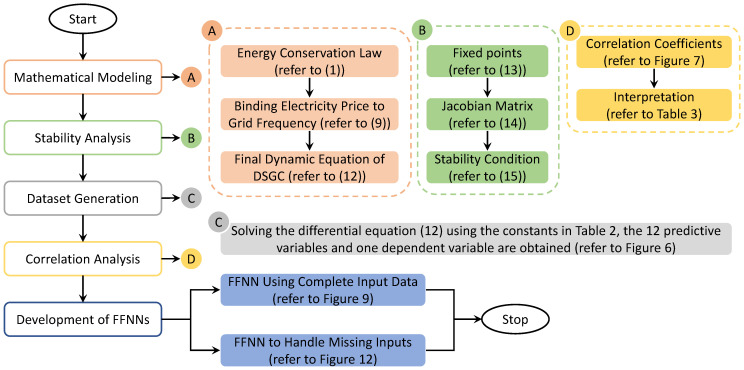
Research flow diagram for the design of smart grid stability model.

A detailed research flowchart of the complete smart grid stability design model is portrayed in Figure 8.

## 4. Development and Performance Evaluation of Feedforward Neural Network

**Figure 9:**
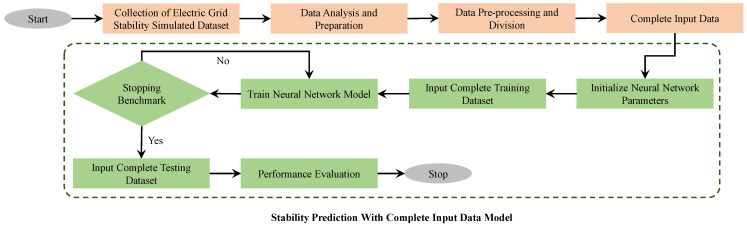
Flow chart of implementation of prediction model with complete input data.

This section develops and evaluates the performance of an FFNN to predict the stability of the smart grid. The methodology for preparing a prediction model using complete input data is shown in Figure 9. The figure shows that data collection, analysis and pre-processing occur first. The input data is identified for the next step, which includes the prediction model to predict stability using the input data. The dataset used in this study consists of 60,000 samples.

**Figure 10:**
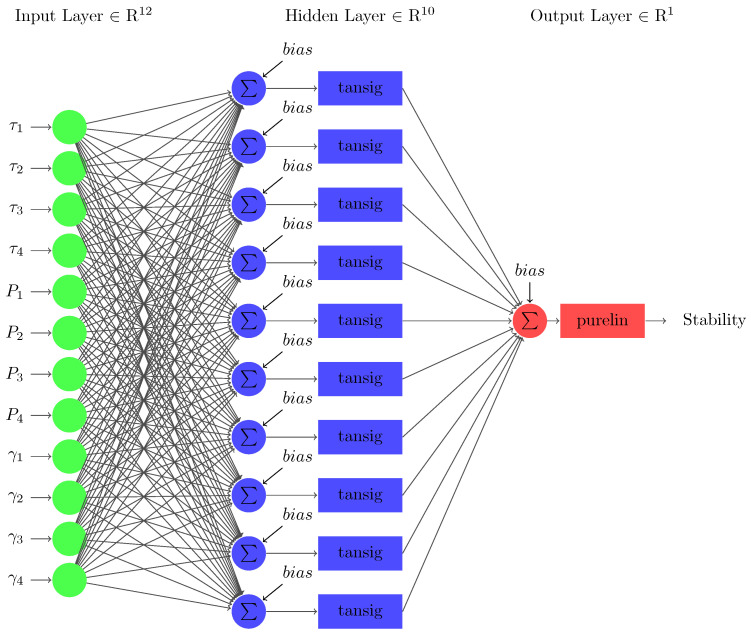
The architecture of FFNN for predicting smart grid’s stability.

The neural network used to predict stability utilizing the input data is a three-layered FFNN as shown in Figure 10. The first layer in the architecture indicates the input layer, which consists of 12 nodes equivalent to the 12 input parameters $\tau_{j}$, $P_{j}$, $\gamma_{j}$∀j∈{1,2,3,4}. The number of nodes in the middle layer, i.e., the hidden layer ‘$N_{h}$,’ is 10, which can be calculated using the number of nodes in input layer ‘$N_{i}$’ as follows [70]:

(17) $$N_{h}=10+\frac{1}{N_{i}}.$$

**Figure 11:**
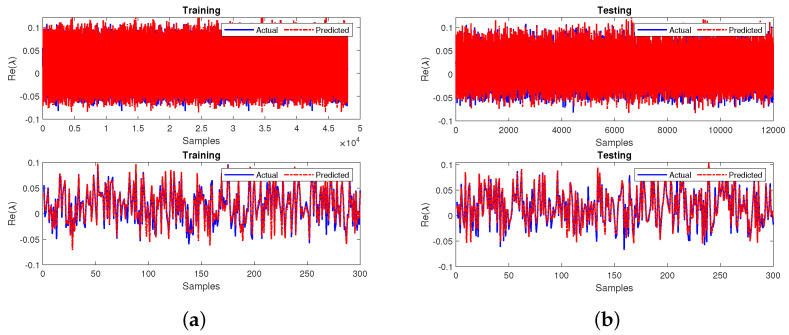
Performance comparison of stability prediction (**a**) training and (**b**) testing.

The third layer represents the output layer, consisting of 1 node, the output parameter (Stability). The dataset is divided into 80% and 20% for training and testing. The neural network is trained using the Levenberg–Marquardt algorithm. The *tansig* and *purelin* activation functions are utilized in the hidden and output layers. The training algorithm and activation functions are chosen as per the results of the comprehensive literature review conducted as shown in Figure 4. The training and testing outputs for the neural network are shown in Figure 11a,b. The neural network performance is measured in terms of R${}^{2}$ and MSE [71,72,73,74]. The model has achieved an R${}^{2}$ value of 0.9739 during training and 0.9738 during testing. Additionally, the model achieved MSE values of 0.0077 during both training and testing. Thereby, the accurate performance of the prediction model is depicted as the R${}^{2}$, and the MSE values are close to 1 and 0.

## 5. Development and Performance Evaluation of Feedforward Neural Network to Handle Missing Input

**Figure 12:**
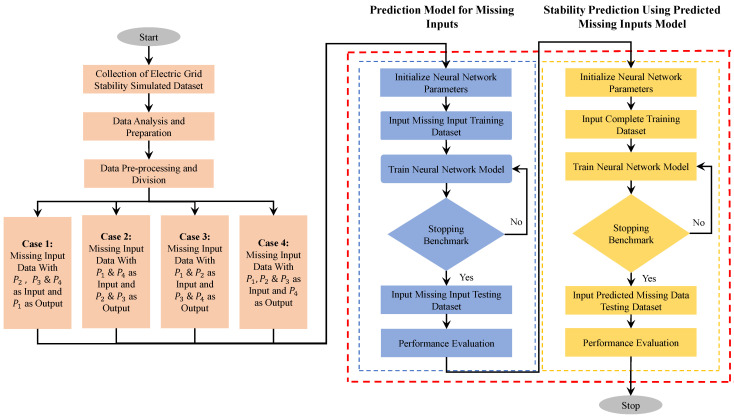
Flow chart of implementation of prediction model that handles missing input data for the four cases.

This section develops and evaluates a novel prediction model to handle missing inputs. The flowchart for the methodology adopted for predicting the missing input data is represented in Figure 12. Herein, four cases of missing inputs are taken, described in the flow chart as Case 1, Case 2, Case 3 and Case 4. This flowchart is represented in three stages.

The first stage includes data collection, analysis, pre-processing and defining the missing inputs. A prediction model is prepared using a sub-neural network to handle the missing inputs in the second stage. After the missing input parameters are predicted, the prediction model that handles missing inputs is prepared to predict stability. The primary neural network is an FFNN trained using the Levenberg–Marquardt algorithm for each case.

The *tansig* and *purelin* transfer functions are used in the hidden and output layers. The dataset consists of 60,000 samples, out of which 80% are used for training and 20% for testing. The input layer consists of 12 nodes corresponding to the 12 input parameters. The output layer consists of one node corresponding to the one output parameter. The number of nodes in the middle layer, i.e., the hidden layer ‘$N_{h}$’, is 10, which can be calculated using (17).

Standard specifications for each sub-neural model in the four cases are as follows: *tansig* and *purelin* transfer functions are used in the hidden and output layers. The dataset, consisting of 60,000 samples, is divided into 80% for training and 20% for testing. The training algorithm used for the sub-neural network is the Levenberg–Marquardt algorithm. Different missing input variables are considered in each layer for each of the four cases, as explained underneath.

### 5.1. Case 1

**Figure 13:**
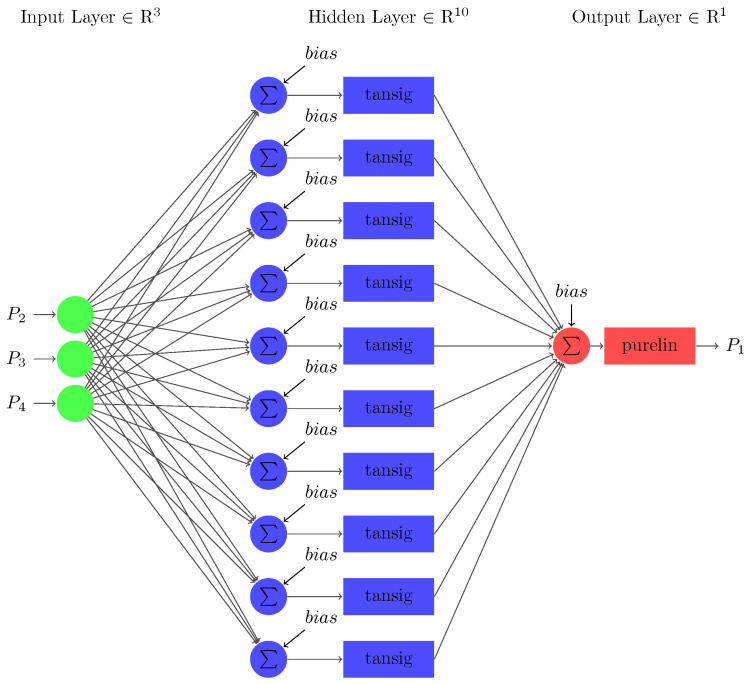
The architecture of FFNN developed for case 1.

One missing input variable is considered in the first case, which will be predicted using a sub-neural network. The sub-neural-network model for this section is an FFNN that consists of three layers (refer to Figure 13). The first input layer consists of three nodes composed of three nodes similar to the three input parameters: accumulated power ($P_{2}$), dissipated power ($P_{3}$) and transmitted power ($P_{4}$). The last layer consists of one output node composed of one output parameter, i.e., source power ($P_{1}$). The number of nodes in the hidden layer is 10, computed using (17).

**Figure 14:**
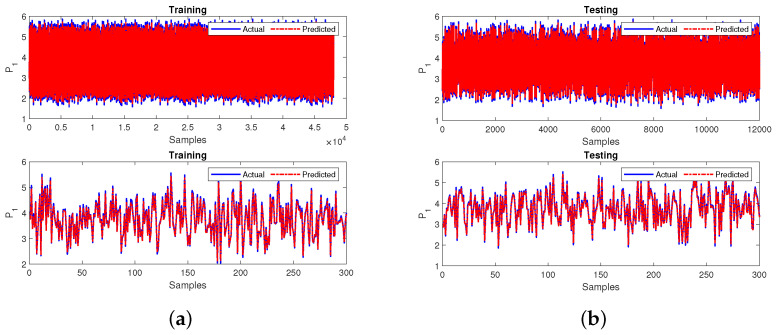
Performance of the sub-neural network for case 1 during (**a**) training and (**b**) testing.

The training and testing outputs for the case 1 sub-neural network is shown in Figure 14a,b for 60,000 samples and zoomed in for 300 samples as shown in the bottom subplot. The neural network performance is measured in terms of R${}^{2}$ and MSE. The model achieved an R${}^{2}$ value of 0.9992 during training and testing. Additionally, the model achieved MSE values of 0.0008 during training and 0.0008 during testing.

**Figure 15:**
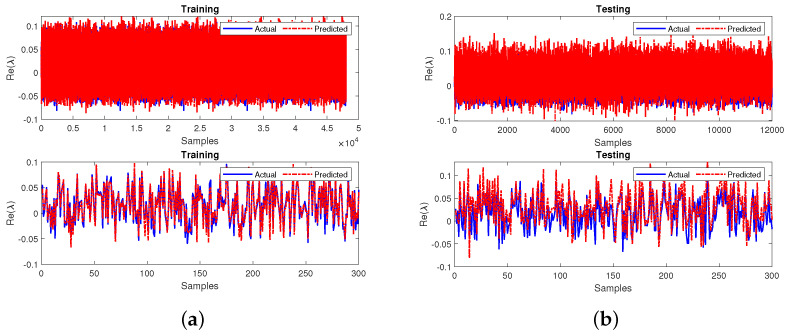
Performance of the primary neural network for case 1 during (**a**) training and (**b**) testing.

The primary neural network was trained to predict stability using the predicted output variables. The testing output variables of the sub-neural network are substituted in the primary neural network. Finally, the leading neural network is tested after predicting the missing input variables. The training and testing outputs for the case 1 primary neural network is shown in Figure 15a,b for 60,000 samples and zoomed in for 300 samples as shown in the bottom subplot. The neural network performance is measured in terms of R${}^{2}$ and MSE. The model achieved an R${}^{2}$ value of 0.9721 during training and 0.8413 during testing. Additionally, the model achieved MSE values of 0.0080 during training and 0.0085 during testing.

### 5.2. Case 2

**Figure 16:**
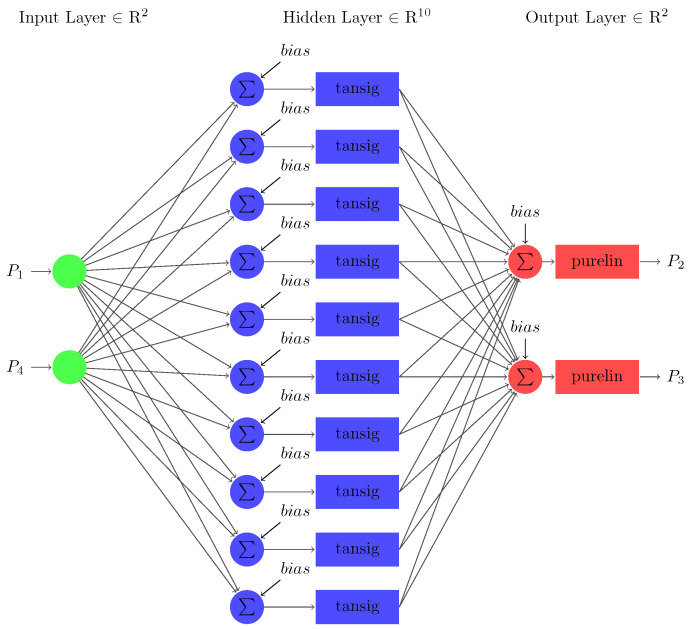
The architecture of FFNN developed for case 2.

Case 2 involves two missing input variables for stability prediction using FFNN as shown in Figure 16. The network shows that the input layer consists of two nodes relative to the input parameters: the source and transmitted powers (i.e., $P_{1}$ and $P_{4}$). The output layer has two nodes corresponding to accumulated and dissipated power (i.e., $P_{2}$ and $P_{3}$). The number of nodes in the hidden layer is 10 (refer to (17)). In the next step, the testing output variables of the sub-neural network are substituted and trained in the primary neural network model.

**Figure 17:**
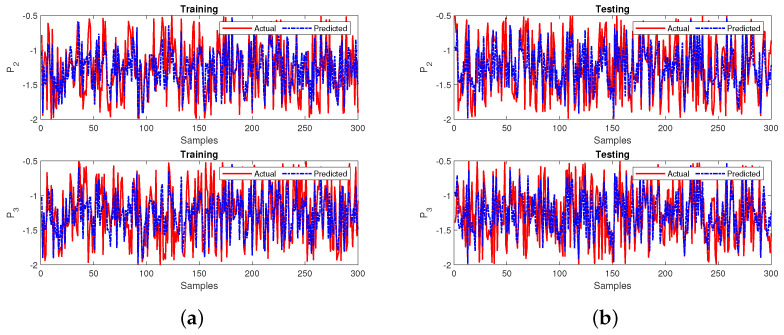
Performance of the sub-neural network for case 2 during (**a**) training and (**b**) testing.

Upon prediction of the missing input variables, the primary neural network is tested. The MSE and R${}^{2}$ performance measures are used to handle the missing data for the prediction model. The sub-neural-network model achieved MSE values of 0.1661 during the training and 0.1667 during testing. The R${}^{2}$ values are 0.7082 during training and 0.7072 during testing. The sub-neural network’s performances during training and testing for the first 300 samples are shown in Figure 17a,b, respectively.

**Figure 18:**
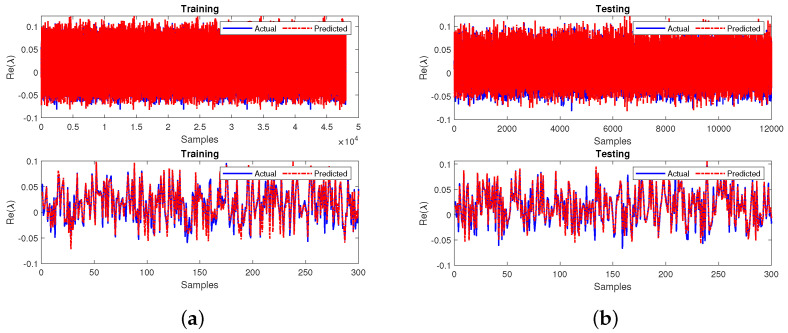
Performance of the primary neural network for case 2 during (**a**) training and (**b**) testing.

The next step of case 2 involves training the primary neural network by utilizing the obtained testing output variables from the sub-neural network that is also measured in terms of MSE and R${}^{2}$ values. The neural network attains an MSE of 0.0077 during training and testing. Furthermore, the R${}^{2}$ values have obtained 0.9738 during the training and testing phases. The final developed model’s performance for all the 60,000 samples and the first 300 samples of both the phases are represented in Figure 18a,b, respectively. The response in plots of the final leading model showcases the best prediction and tracking ability at both phases. The MSE and R${}^{2}$ values relative to 0 and 1, respectively, indicate that the final proposed model for this case gives a superior performance.

### 5.3. Case 3

**Figure 19:**
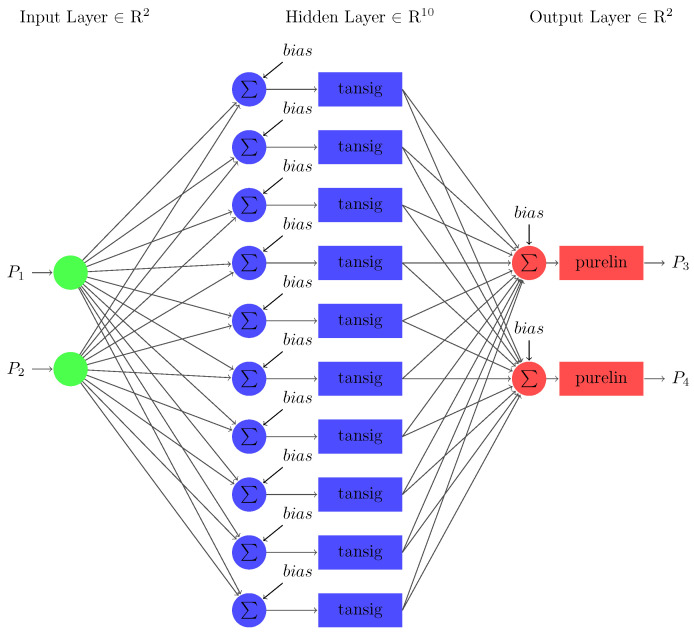
The architecture of FFNN developed for case 3.

Further, Case 3 uses two missing input variables for a feedforward sub-neural network for stability prediction as shown in Figure 19. The input layer has two nodes relative to the two input parameters: the source power and accumulated power (i.e., $P_{1}$ and $P_{2}$). The output layer has two nodes corresponding to the two output parameters, dissipated and transmitted powers (i.e., $P_{3}$ and $P_{4}$). The number of nodes in the hidden layer is 10. In the next step, the testing output variables of the sub-neural network are substituted and trained in the primary neural network model. Upon prediction of the missing input variables, the primary neural network is trained. The MSE and R${}^{2}$ performance measures are used to handle the missing data for the prediction model. The sub-neural-network model achieved MSE values of 0.1659 during the training and 0.1673 during the testing phases.

**Figure 20:**
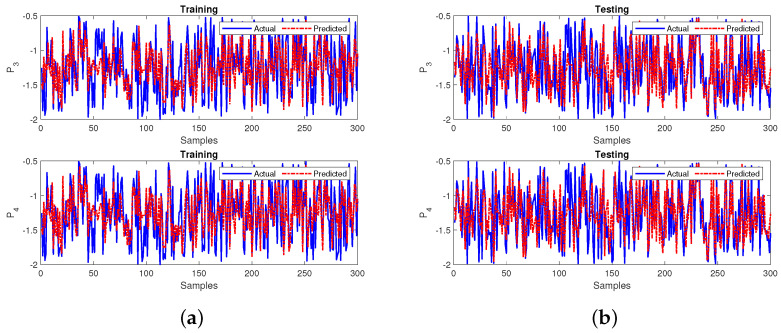
Performance of the sub-neural network for case 3 during (**a**) training and (**b**) testing.

The R${}^{2}$ values are found to be 0.7085 and 0.7061 during training and testing. The sub-neural network’s performance during training and testing for the first 300 samples is shown in Figure 20a,b, respectively.

**Figure 21:**
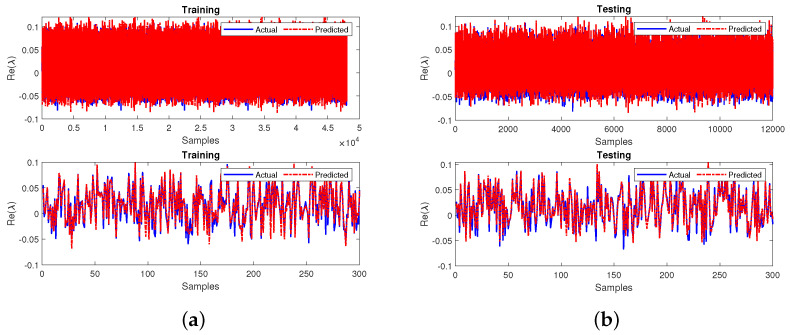
Performance of the primary neural network for case 3 during (**a**) training and (**b**) testing.

Next, the primary neural network was trained by utilizing the obtained testing output variables from the sub-neural network measured using MSE and R${}^{2}$ values. The neural network attained an MSE of 0.0083 during training and 0.0082 during testing. Furthermore, the R${}^{2}$ values obtained were 0.9720 during training and 0.9721 during the testing phases. The final developed model’s performance for the 60,000 samples and the first 300 zoomed-in samples at both stages are represented in Figure 21a,b, respectively. The response in plots of the final leading model showcases the best prediction and tracking ability at both phases. The MSE and R${}^{2}$ values relative to 0 and 1, respectively, indicate that the final proposed model for this case gives a satisfactory performance.

### 5.4. Case 4

**Figure 22:**
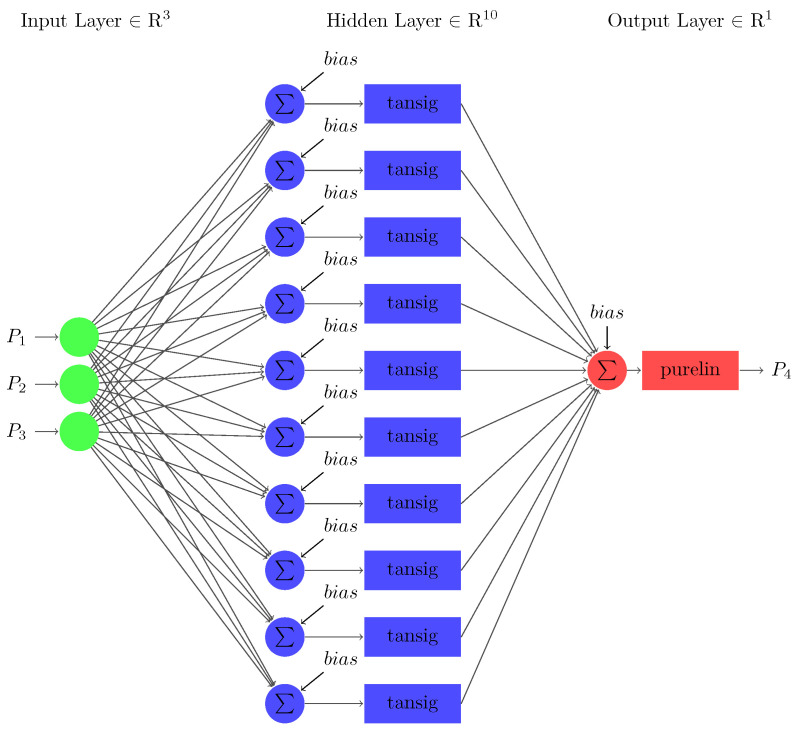
The architecture of FFNN developed for case 4.

Finally, the study of case 4 considers one missing input variable that is predicted using a sub-neural network as shown in Figure 22. Here, the first input layer has three nodes representing the input parameters: the source power, accumulated power and dissipated power (i.e., $P_{1}$, $P_{2}$ and $P_{3}$). The last layer, the output layer, is composed of one node corresponding to the transmitted power (i.e., $P_{4}$). The number of nodes in the hidden layer is 10. The primary neural network was trained to use the predicted output variables to predict the stability after replacement in the primary neural network.

**Figure 23:**
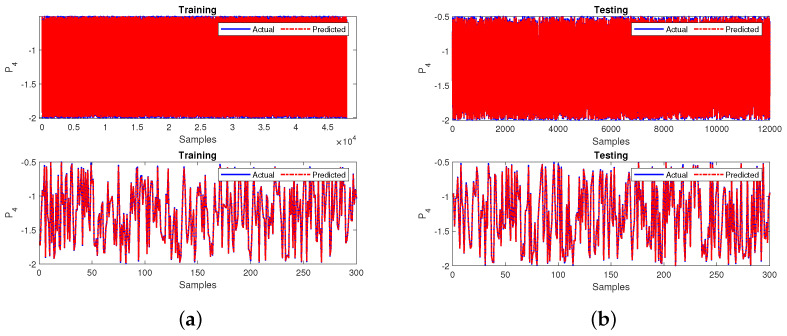
Performance of the sub-neural network for case 4 during (**a**) training and (**b**) testing.

Once the missing input variable is predicted, the primary neural network is trained similarly to previous cases. The model’s performance handling the missing data is measured using R${}^{2}$ and MSE. The sub-neural-network model achieved MSE values are 0.0001 in during training and testing and an R${}^{2}$ value of 0.9999 during the training and testing phases. The performance of the sub-neural-network model developed for all the 60,000 samples and the first 300 samples zoomed in is depicted in Figure 23a,b for training and testing, respectively.

**Figure 24:**
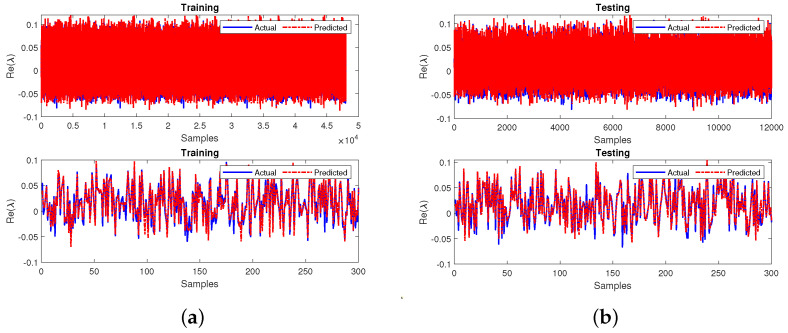
Performance of the primary neural network for case 4 during (**a**) training and (**b**) testing.

Further, the primary neural network was trained similarly to other cases by utilizing the testing output variables from the sub-neural network. The model obtains an MSE of 0.0084 during training and 0.0084 during the testing phase. The R${}^{2}$ values were 0.9717 during training and 0.9715 during testing. The performance of the final developed neural network having 60,000 samples and the first 300 samples during both training and testing are shown in Figure 24a,b, respectively. The model’s response offers both phases the best training and prediction ability. The MSE values obtained are close to 0, and the R${}^{2}$ values are relative to 1, highlighting that the proposed model is accurate for stability prediction.

### 5.5. Summary

Table 4 depicts the performance evaluation of the developed FFNN model using complete input data and the model that handles the missing inputs (Case 1, Case 2, Case 3 and Case 4). The R${}^{2}$ and MSE results of the sub-neural-network model of case 4, with one missing input variable: transmitted power (i.e., $P_{4}$), shows the model’s best training and prediction ability in both phases. For all the sub-neural-network models, the R${}^{2}$ value has achieved at least 70% and 97% for the primary neural network. We noticed that the MSE values obtained are close to 0. Furthermore, the R${}^{2}$ values are relative to 1, which indicates the excellent performance of all the models.

## 6. Conclusions

The primary goal of this paper was to tackle the issue of stability prediction when there are missing variables involved. This missing variable could be due to the failure of a sensor, network connection or other system. This paper successfully solved this issue by proposing a novel FFNN model that handles missing inputs. The model’s performance was evaluated on a four-node star network. In this study, four cases of missing input variables were taken.

For each case, a sub-neural network was first prepared to predict the missing variables, and then these predicted values were fed into the primary neural network to predict the stability. Among all four cases, case 4 showed the best performance with an MSE value of 0.0001 and an R${}^{2}$ value of 0.9999 during training and testing for the sub-neural network. In addition, the primary network showed an MSE value of 0.0084 and an R${}^{2}$ value of 0.9717 during training and 0.9715 during testing. For all the four cases, the models achieved an MSE close to 0 and an R${}^{2}$ value close to 1 thus indicating the excellent performance of the prediction models.

However, this work was limited to predicting the power parameter using a sub-neural network because the algebraic sum of the power consumed or generated was assumed to be zero, and uncertainties and disturbances were not considered. Moreover, the reaction time and price elasticity parameters are highly nonlinear in the considered dataset. As a result, this proposed model faces a shortcoming in predicting the missing variables (reaction time and price elasticity) using a sub-neural network that predicts the stability using a primary network. Therefore, extending the proposed model to predict these highly nonlinear input parameters will be addressed in our future work.

## Figures and Tables

**Table 2:** Predictive features and simulation constants used for data generation of four-node star network.

Category	Parameter	Range/Value
	$\tau_{j}$	[0.5,10]s
Predictive features	$P_{j}$	$\left[-2.0,-0.5\right]\;s^{-2}$
	$\gamma_{j}$	$\left[0.05,1\right]\;s^{-1}$
	$\alpha_{j}$	$0.1\;s^{-1}$
Simulation constants	$T_{j}$	2s
	$K_{jm}$	$8\;s^{-2}$

**Table 3:** Interpretation of Pearson’s correlation coefficients.

Coefficient	Interpretation
±0.90–±1.00	Very strong correlation
±0.70–±0.89	Strong correlation
±0.40–±0.69	Moderate correlation
±0.10–±0.39	Weak correlation
0.00–±0.09	Negligible correlation

**Table 4:** Performance evaluation comparison of the FFNN with complete input data and the FFNN that handles the missing data.

Category	Case	Network	Stage	R${}^{2}$	MSE
With Complete Input Data	-	Primary	Training	0.9739	0.0077
			Testing	0.9738	0.0077
Model that Handles Missing Input Data	Case 1	Sub	Training	0.9992	0.0008
			Testing	0.9992	0.0008
		Primary	Training	0.9721	0.0080
			Testing	0.8413	0.0085
	Case 2	Sub	Training	0.7082	0.1661
			Testing	0.7072	0.1667
		Primary	Training	0.9738	0.0077
			Testing	0.9738	0.0077
	Case 3	Sub	Training	0.7085	0.1659
			Testing	0.7061	0.1673
		Primary	Training	0.9720	0.0083
			Testing	0.9721	0.0082
	Case 4	Sub	Training	0.9999	0.0001
			Testing	0.9999	0.0001
		Primary	Training	0.9717	0.0084
			Testing	0.9715	0.0084

## Data Availability

The data used in this research are available from the UC Irvine Machine Learning Repository (https://archive.ics.uci.edu/ml/datasets/Electrical+Grid+Stability+Simulated+Data+, accessed on 15 February 2022).

## Abbreviations

The following abbreviations are used in this manuscript:

AdaBoost	Adaptive Boosting
AdaGrad	Adaptive Gradient algorithm
Adam	Adaptive Movement Estimation
ACE	Average Coverage Error
Adaptive LR	Adaptive Linear Regression
AFC	Accurate and Fast Converging
ANN	Artificial Neural Network
ARIMA	Autoregressive Integrated Moving Average
ARMA	Autoregressive Moving Average
ARMAX	Autoregressive-Moving-Average Model With Exogenous Inputs
BiGRU	Bidirectional Gated Recurrent Unit
BP	Back Propagation
BPNN	Back Propagation Neural Network
BR	Bayesian Regularization
C-DDPG	Centralized Based Deep Deterministic Policy Gradient
CDF	Cumulative Distribution Function
CEEMDAN	Complete Ensemble Empirical Mode Decomposition Adaptive Noise
CGA	Chaos Search Genetic Algorithm
CGASA	Chaos Search Genetic Algorithm Furthermore, Simulated Annealing
CNN	Convolutional Neural Network
CRBM	Convolutional Restricted Boltzmann Machine
DBN	Deep Belief Network
DNN	Deep Neural Network
DPCS	Distributed Power Consumption Scheduling
DSGC	Decentral Smart Grid Control
DT	Decision Tree
ECNN	Enhanced Convolutional Neural Network
ELM	Extreme Learning Machine
ENN	Elman Neural Network
ESS	Energy Storage Systems
FA	Forecast Accuracy
FF	Feedforward
FFNN	Feedforward Neural Network
FS	Forecast Skill
GA	Genetic Algorithm
GBR	Gradient Boosting Regression
GDM	Gradient Descent Method
GRU	Gated Recurrent Unit
HR	Hit Rate
IBR	Inclining Block Rate
IRBDNN	Iterative Resblock Based Deep Neural Network
KNN	k-Nearest Neighbors
LBPP	Load Based Pricing Policy
LGBM	Light Gradient Boosting Machine
LIF	Leaky Integrate and Fire Neuron
LM	Levenberg–Marquardt
LMS	Lagrange Multiplier Selection
LQE	Link Quality Estimation
LSSVR	Least Squares Support Vector Regression
LVQ	Learning Vector Quantization
MABE	Mean Absolute Bias Error
MAD	Median Absolute Deviation
MAE	Mean Absolute Error
MAPE	Mean Absolute Percentage Error
MBE	Mean Biased Error
MCC	Matthews Correlation Coefficient
MdBP	Modified Back Propagation
MER	Mean Error Rate
MI-ANN	Mutual Information Artificial Neural Network
MLP	Multi-Layer Perceptron
MLR	Multi-variable Linear Regression
MLSTM	Multiplicative Long Short-Term Memory
MNE	Mean Normalized Error
MPE	Mean Percentage Error
MSE	Mean Square Error
Nadam	Nesterov-accelerated Adaptive Moment Estimation
NARX	Nonlinear Autoregressive Network With Exogenous Inputs
NN	Neural Network
NRMSE	Normalized Root Mean Square Error
PAR	Peak To Average Ratio
PDF	Probability Density Function
PDNN	Pooling Based Deep Neural Network
PICP	Prediction Interval Coverage Probability
PINC	Prediction Interval Nominal Confidence
PSO	Particle Swarm Optimization
PTECC	Proportion Of Total Energy Classified Correctly
PV	Photo Voltaic
R	Correlation Coefficient
RBF	Radial Basis Function
RBFNN	Based Radial Basis Function
RDNN	Recurrent Deep Neural Network
RE	Relative Error
ReLU	Rectified Linear Activation Unit
RES	Renewable Energy Sources
RF	Random Forest
RL	Reinforcement Learning
RMSE	Root Mean Square Error
RNN	Recurrent Neural Network
RTEP	Real Time Electrical Pricing
RTP	Real Time Price
SAE	Sparse Auto Encoder
SCG	Scaled Conjugate Gradient
SMP	Spot Market Price
SNN	Spiking Neural Network
SNR	Signal to Noise Ratio
SoC	Speed of Convergence
SPREAD	Spread of Radial Basis Functions
SSA	Salp Swam Algorithm
STLF	Short Term Load Forecasting
STW	Sliding Time Window
SVM	Support Vector Machine
SWAA	Sample Weighted Average Approximation
Tanh	Hyperbolic Tangent Function
WNN	Wavelet Neural Network
WOA	Whale Optimization Algorithm
WRNN	Wavelet Recurrent Neural Network
XGB	Extreme Gradient Boosting

## References

[1] Gharavi H., Ghafurian R. (2011). Smart Grid: The Electric Energy System of the Future.

[2] McLaughlin K., Friedberg I., Kang B., Maynard P., Sezer S., McWilliams G. (2015). Secure communications in smart grid: Networking and protocols. Smart Grid Security.

[3] Rathnayaka A.D., Potdar V.M., Dillon T., Kuruppu S. (2015). Framework to manage multiple goals in community-based energy sharing network in smart grid. Int. J. Electr. Power Energy Syst..

[4] Breviglieri P., Erdem T., Eken S. (2021). Predicting Smart Grid Stability with Optimized Deep Models. SN Comput. Sci..

[5] Schäfer B., Grabow C., Auer S., Kurths J., Witthaut D., Timme M. (2016). Taming instabilities in power grid networks by decentralized control. Eur. Phys. J. Spec. Top..

[6] Verma K., Niazi K. Generator coherency determination in a smart grid using artificial neural network. Proceedings of the 2012 IEEE Power and Energy Society General Meeting.

[7] Karthikumar K., Karthik K., Karunanithi K., Chandrasekar P., Sathyanathan P., Prakash S.V.J. (2021). SSA-RBFNN strategy for optimum framework for energy management in Grid-Connected smart grid infrastructure modeling. Mater. Today Proc..

[8] Massaoudi M., Abu-Rub H., Refaat S.S., Chihi I., Oueslati F.S. Accurate Smart-Grid Stability Forecasting Based on Deep Learning: Point and Interval Estimation Method. Proceedings of the 2021 IEEE Kansas Power and Energy Conference (KPEC).

[9] Xia M., Shao H., Ma X., de Silva C.W. (2021). A Stacked GRU-RNN-based Approach for Predicting Renewable Energy and Electricity Load for Smart Grid Operation. IEEE Trans. Ind. Inform..

[10] Gao B., Huang X., Shi J., Tai Y., Zhang J. (2020). Hourly forecasting of solar irradiance based on CEEMDAN and multi-strategy CNN-LSTM neural networks. Renew. Energy.

[11] Li J., Deng D., Zhao J., Cai D., Hu W., Zhang M., Huang Q. (2021). A novel hybrid short-term load forecasting method of smart grid using mlr and lstm neural network. IEEE Trans. Ind. Inform..

[12] Mohammad F., Kim Y.C. (2020). Energy load forecasting model based on deep neural networks for smart grids. Int. J. Syst. Assur. Eng. Manag..

[13] Capizzi G., Sciuto G.L., Napoli C., Tramontana E. (2018). Advanced and adaptive dispatch for smart grids by means of predictive models. IEEE Trans. Smart Grid.

[14] Jeyaraj P.R., Nadar E.R.S. (2021). Computer-assisted demand-side energy management in residential smart grid employing novel pooling deep learning algorithm. Int. J. Energy Res..

[15] Islam B., Baharudin Z., Nallagownden P. (2017). Development of chaotically improved meta-heuristics and modified BP neural network-based model for electrical energy demand prediction in smart grid. Neural Comput. Appl..

[16] Gupta S., Kazi F., Wagh S., Kambli R. (2015). Neural network based early warning system for an emerging blackout in smart grid power networks. Intelligent Distributed Computing.

[17] Neupane B., Perera K.S., Aung Z., Woon W.L. Artificial neural network-based electricity price forecasting for smart grid deployment. Proceedings of the 2012 International Conference on Computer Systems and Industrial Informatics.

[18] Verma K., Niazi K. Determination of vulnerable machines for online transient security assessment in smart grid using artificial neural network. Proceedings of the 2011 Annual IEEE India Conference.

[19] Sakellariou M., Ferentinou M. (2005). A study of slope stability prediction using neural networks. Geotech. Geol. Eng..

[20] Tu J.V. (1996). Advantages and disadvantages of using artificial neural networks versus logistic regression for predicting medical outcomes. J. Clin. Epidemiol..

[21] Nijman S., Leeuwenberg A., Beekers I., Verkouter I., Jacobs J., Bots M., Asselbergs F., Moons K., Debray T. (2022). Missing data is poorly handled and reported in prediction model studies using machine learning: A literature review. J. Clin. Epidemiol..

[22] Wang S., Bi S., Zhang Y.J.A. (2020). Locational detection of the false data injection attack in a smart grid: A multilabel classification approach. IEEE Internet Things J..

[23] Niu X., Li J., Sun J., Tomsovic K. Dynamic detection of false data injection attack in smart grid using deep learning. Proceedings of the 2019 IEEE Power & Energy Society Innovative Smart Grid Technologies Conference (ISGT).

[24] Sun W., Lu W., Li Q., Chen L., Mu D., Yuan X. (2017). WNN-LQE: Wavelet-neural-network-based link quality estimation for smart grid WSNs. IEEE Access.

[25] Ungureanu S., Ţopa V., Cziker A. Integrating the industrial consumer into smart grid by load curve forecasting using machine learning. Proceedings of the 2019 8th International Conference on Modern Power Systems (MPS).

[26] Alamaniotis M. Synergism of deep neural network and elm for smart very-short-term load forecasting. Proceedings of the 2019 IEEE PES Innovative Smart Grid Technologies Europe (ISGT-Europe).

[27] Zahid M., Ahmed F., Javaid N., Abbasi R.A., Zainab Kazmi H.S., Javaid A., Bilal M., Akbar M., Ilahi M. (2019). Electricity price and load forecasting using enhanced convolutional neural network and enhanced support vector regression in smart grids. Electronics.

[28] Çavdar İ.H., Faryad V. (2019). New design of a supervised energy disaggregation model based on the deep neural network for a smart grid. Energies.

[29] Selim M., Zhou R., Feng W., Quinsey P. (2021). Estimating Energy Forecasting Uncertainty for Reliable AI Autonomous Smart Grid Design. Energies.

[30] Alazab M., Khan S., Krishnan S.S.R., Pham Q.V., Reddy M.P.K., Gadekallu T.R. (2020). A multidirectional LSTM model for predicting the stability of a smart grid. IEEE Access.

[31] Hasan M., Toma R.N., Nahid A.A., Islam M., Kim J.M. (2019). Electricity theft detection in smart grid systems: A CNN-LSTM based approach. Energies.

[32] Xu B., Guo F., Zhang W.A., Li G., Wen C. (2021). E2DNet: An Ensembling Deep Neural Network for Solving Nonconvex Economic Dispatch in Smart Grid. IEEE Trans. Ind. Inform..

[33] Hong Y., Zhou Y., Li Q., Xu W., Zheng X. (2020). A deep learning method for short-term residential load forecasting in smart grid. IEEE Access.

[34] Zheng Y., Celik B., Suryanarayanan S., Maciejewski A.A., Siegel H.J., Hansen T.M. (2021). An aggregator-based resource allocation in the smart grid using an artificial neural network and sliding time window optimization. IET Smart Grid.

[35] Bingi K., Prusty B.R. (2021). Forecasting models for chaotic fractional-order oscillators using neural networks. Int. J. Appl. Math. Comput. Sci..

[36] Bingi K., Prusty B.R. Chaotic Time Series Prediction Model for Fractional-Order Duffing’s Oscillator. Proceedings of the 2021 8th International Conference on Smart Computing and Communications (ICSCC).

[37] Bingi K., Prusty B.R. Neural Network-Based Models for Prediction of Smart Grid Stability. Proceedings of the 2021 Innovations in Power and Advanced Computing Technologies (i-PACT).

[38] Qi X., Chen G., Li Y., Cheng X., Li C. (2019). Applying neural-network-based machine learning to additive manufacturing: Current applications, challenges, and future perspectives. Engineering.

[39] Sattari M.A., Roshani G.H., Hanus R., Nazemi E. (2021). Applicability of time-domain feature extraction methods and artificial intelligence in two-phase flow meters based on gamma-ray absorption technique. Measurement.

[40] Chandrasekaran K., Selvaraj J., Amaladoss C.R., Veerapan L. (2021). Hybrid renewable energy based smart grid system for reactive power management and voltage profile enhancement using artificial neural network. Energy Sources Part A Recover. Util. Environ. Eff..

[41] Zhou Z., Xiang Y., Xu H., Wang Y., Shi D. (2021). Unsupervised Learning for Non-Intrusive Load Monitoring in Smart Grid Based on Spiking Deep Neural Network. J. Mod. Power Syst. Clean Energy.

[42] Chung H.M., Maharjan S., Zhang Y., Eliassen F. (2021). Distributed deep reinforcement learning for intelligent load scheduling in residential smart grids. IEEE Trans. Ind. Inform..

[43] Cahyono M.R.A. Design Power Controller for Smart Grid System Based on Internet of Things Devices and Artificial Neural Network. Proceedings of the 2020 IEEE International Conference on Internet of Things and Intelligence System (IoTaIS).

[44] Khan S., Kifayat K., Kashif Bashir A., Gurtov A., Hassan M. (2021). Intelligent intrusion detection system in smart grid using computational intelligence and machine learning. Trans. Emerg. Telecommun. Technol..

[45] Ullah A., Javaid N., Samuel O., Imran M., Shoaib M. CNN and GRU based deep neural network for electricity theft detection to secure smart grid. Proceedings of the 2020 International Wireless Communications and Mobile Computing (IWCMC).

[46] Ruan G., Zhong H., Wang J., Xia Q., Kang C. (2020). Neural-network-based Lagrange multiplier selection for distributed demand response in smart grid. Appl. Energy.

[47] Di Piazza A., Di Piazza M.C., La Tona G., Luna M. (2021). An artificial neural network-based forecasting model of energy-related time series for electrical grid management. Math. Comput. Simul..

[48] Khalid Z., Abbas G., Awais M., Alquthami T., Rasheed M.B. (2020). A novel load scheduling mechanism using artificial neural network based customer profiles in smart grid. Energies.

[49] Fan L., Li J., Pan Y., Wang S., Yan C., Yao D. Research and application of smart grid early warning decision platform based on big data analysis. Proceedings of the 2019 4th International Conference on Intelligent Green Building and Smart Grid (IGBSG).

[50] Li G., Wang H., Zhang S., Xin J., Liu H. (2019). Recurrent neural networks based photovoltaic power forecasting approach. Energies.

[51] Huang X., Shi J., Gao B., Tai Y., Chen Z., Zhang J. (2019). Forecasting hourly solar irradiance using hybrid wavelet transformation and Elman model in smart grid. IEEE Access.

[52] Haghnegahdar L., Wang Y. (2020). A whale optimization algorithm-trained artificial neural network for smart grid cyber intrusion detection. Neural Comput. Appl..

[53] Ahmed F., Zahid M., Javaid N., Khan A.B.M., Khan Z.A., Murtaza Z. (2019). A deep learning approach towards price forecasting using enhanced convolutional neural network in smart grid. International Conference on Emerging Internetworking, Data & Web Technologies.

[54] Duong-Ngoc H., Nguyen-Thanh H., Nguyen-Minh T. Short term load forcast using deep learning. Proceedings of the 2019 Innovations in Power and Advanced Computing Technologies (i-PACT).

[55] Kulkarni S.N., Shingare P. Artificial Neural Network Based Short Term Power Demand Forecast for Smart Grid. Proceedings of the 2018 IEEE Conference on Technologies for Sustainability (SusTech).

[56] Ghasemi A.A., Gitizadeh M. (2018). Detection of illegal consumers using pattern classification approach combined with Levenberg-Marquardt method in smart grid. Int. J. Electr. Power Energy Syst..

[57] Vrablecová P., Ezzeddine A.B., Rozinajová V., Šárik S., Sangaiah A.K. (2017). Smart grid load forecasting using online support vector regression. Comput. Electr. Eng..

[58] Ahmad A., Javaid N., Guizani M., Alrajeh N., Khan Z.A. (2017). An accurate and fast converging short-term load forecasting model for industrial applications in a smart grid. IEEE Trans. Ind. Inform..

[59] Li L., Ota K., Dong M. Everything is image: CNN-based short-term electrical load forecasting for smart grid. Proceedings of the 2017 14th International Symposium on Pervasive Systems, Algorithms and Networks & 2017 11th International Conference on Frontier of Computer Science and Technology & 2017 Third International Symposium of Creative Computing (ISPAN-FCST-ISCC).

[60] Bicer Y., Dincer I., Aydin M. (2016). Maximizing performance of fuel cell using artificial neural network approach for smart grid applications. Energy.

[61] Macedo M.N., Galo J.J., De Almeida L., Lima A.d.C. (2015). Demand side management using artificial neural networks in a smart grid environment. Renew. Sustain. Energy Rev..

[62] Muralidharan S., Roy A., Saxena N. Stochastic hourly load forecasting for smart grids in korea using narx model. Proceedings of the 2014 International Conference on Information and Communication Technology Convergence (ICTC).

[63] Ioakimidis C., Eliasstam H., Rycerski P. Solar power forecasting of a residential location as part of a smart grid structure. Proceedings of the 2012 IEEE Energytech.

[64] Hashiesh F., Mostafa H.E., Khatib A.R., Helal I., Mansour M.M. (2012). An intelligent wide area synchrophasor based system for predicting and mitigating transient instabilities. IEEE Trans. Smart Grid.

[65] Fei W., Zengqiang M., Shi S., Chengcheng Z. A practical model for single-step power prediction of grid-connected PV plant using artificial neural network. Proceedings of the 2011 IEEE PES Innovative Smart Grid Technologies.

[66] Qudaih Y.S., Mitani Y. (2011). Power distribution system planning for smart grid applications using ANN. Energy Procedia.

[67] Zhang H.T., Xu F.Y., Zhou L. Artificial neural network for load forecasting in smart grid. Proceedings of the 2010 International Conference on Machine Learning and Cybernetics.

[68] Arzamasov V., Böhm K., Jochem P. Towards concise models of grid stability. Proceedings of the 2018 IEEE International Conference on Communications, Control, and Computing Technologies for Smart Grids (SmartGridComm).

[69] Akoglu H. (2018). User’s guide to correlation coefficients. Turk. J. Emerg. Med..

[70] Sheela K.G., Deepa S.N. (2013). Review on methods to fix number of hidden neurons in neural networks. Math. Probl. Eng..

[71] Bingi K., Prusty B.R., Panda K.P., Panda G. (2022). Time Series Forecasting Model for Chaotic Fractional-Order Rössler System. Sustainable Energy and Technological Advancements.

[72] Shaik N.B., Pedapati S.R., Othman A., Bingi K., Dzubir F.A.A. (2021). An intelligent model to predict the life condition of crude oil pipelines using artificial neural networks. Neural Comput. Appl..

[73] Bingi K., Prusty B.R., Kumra A., Chawla A. Torque and temperature prediction for permanent magnet synchronous motor using neural networks. Proceedings of the 2020 3rd International Conference on Energy, Power and Environment: Towards Clean Energy Technologies.

[74] Ramadevi B., Bingi K. (2022). Chaotic Time Series Forecasting Approaches Using Machine Learning Techniques: A Review. Symmetry.

